# Effects of the Clock Modulator Nobiletin on Circadian Rhythms and Pathophysiology in Female Mice of an Alzheimer’s Disease Model

**DOI:** 10.3390/biom11071004

**Published:** 2021-07-09

**Authors:** Eunju Kim, Kazunari Nohara, Marvin Wirianto, Gabriel Escobedo, Ji Ye Lim, Rodrigo Morales, Seung-Hee Yoo, Zheng Chen

**Affiliations:** 1 Department of Biochemistry and Molecular Biology, The University of Texas Health Science Center at Houston, Houston, TX 77030, USA; Eunju.Kim@uth.tmc.edu (E.K.); Kazunari.Nohara@bcm.edu (K.N.); Marvin.Wirianto@uth.tmc.edu (M.W.); JiYe.Lim@uth.tmc.edu (J.Y.L.); Seung-Hee.Yoo@uth.tmc.edu (S.-H.Y.); 2 Department of Neurology, The University of Texas Health Science Center at Houston, Houston, TX 77030, USA; Gabriel.Escobedo@bcm.edu (G.E.J.); Rodrigo.MoralesLoyola@uth.tmc.edu (R.M.); 3 Centro Integrativo de Biologia y Química Aplicada (CIBQA), Universidad Bernardo O’Higgins, Santiago 8370993, Chile

**Keywords:** Alzheimer’s disease, female APP/PS1 mice, Nobiletin (NOB), circadian rhythms, sleep, energy metabolism, mitochondria, amyloid beta (Aβ)

## Abstract

Alzheimer’s disease (AD) is an age-related neurodegenerative disorder and the most common cause of dementia. Various pathogenic mechanisms have been proposed to contribute to disease progression, and recent research provided evidence linking dysregulated circadian rhythms/sleep and energy metabolism with AD. Previously, we found that the natural compound Nobiletin (NOB) can directly activate circadian cellular oscillators to promote metabolic health in disease models and healthy aging in naturally aged mice. In the current study, using the amyloid-β AD model APP/PS1, we investigated circadian, metabolic and amyloid characteristics of female mice and the effects of NOB. Female APP/PS1 mice showed reduced sleep bout duration, and NOB treatment exhibited a trend to improve it. While glucose tolerance was unchanged, female APP/PS1 mice displayed exaggerated oxygen consumption and CO2 production, which was mitigated by NOB. Likewise, cold tolerance in APP/PS1 was impaired relative to WT, and interestingly was markedly enhanced in NOB-treated APP/PS1 mice. Although circadian behavioral rhythms were largely unchanged, real-time qPCR analysis revealed altered expression of several core clock genes by NOB in the cerebral cortex, notably *Bmal1*, *Npas2*, and *Rora*. Moreover, NOB was also able to activate various clock-controlled metabolic genes involved in insulin signaling and mitochondrial function, including *Igf1*, *Glut1*, *Insr*, *Irs1*, *Ucp2*, and *Ucp4*. Finally, we observed that NOB attenuated the expression of several AD related genes including *App*, *Bace1*, and *ApoE*, reduced APP protein levels, and strongly ameliorated Aβ pathology in the cortex. Collectively, these results reveal novel genotype differences and importantly beneficial effects of a natural clock-enhancing compound in biological rhythms and related pathophysiology, suggesting the circadian clock as a modifiable target for AD.

## 1. Introduction

Alzheimer’s disease is the most common form of dementia affecting the aged population, with pathological hallmarks of extracellular amyloid-β (Aβ) plaques and intracellular neurofibrillary tau tangles [1]. The vast majority of human AD cases are sporadic in nature, with years and decades between first emergence of pathological changes and clinical manifestation. Therefore, a number of pathophysiological processes have been proposed to contribute to its pathogenesis. In recent years, dysregulated circadian/sleep cycles have emerged as a contributory mechanism for AD [2,3,4,5]. While aging is known to correlate with sleep fragmentation and reduced amplitude (e.g., shorter bout duration), sleep in AD patients is even more severely disrupted, accompanied by nocturnal activity/daytime sleepiness, eventually deteriorating into almost reversed cycles which requires institutionalization [4]. A number of studies have reported that extracellular Aβ and Tau abundance undergoes diurnal fluctuation which is altered by sleep deprivation and orexin drugs that promote sleep [6,7,8,9,10]. Importantly, human studies have shown that sleep fragmentation or perturbed daily activity patterns first arise during the pre-symptomatic phase of AD, which can be used as a marker to predict cognitive deficits, pathological Aβ deposition and dementias [11,12].

The circadian clock plays a key role in sleep regulation [13,14]. In mammals, the clock consists of cell-autonomous molecular oscillators containing positive (CLOCK, NPAS2, BMAL1, RORs) and negative (PERs, CRYs, REV-ERBs) factors organized into interlocked transcription/translation feedback loops [15]. These oscillators are synchronized by the master pacemaker located in the suprachiasmatic nuclei (SCN) in the hypothalamus, primarily functioning to control gene expression and downstream physiological functions throughout the body [16,17]. For example, genomic and molecular studies have revealed oscillatory expression and clock regulation of key genes involved in AD, including *Bace1* [8,18,19]. While brains from AD patients have also been shown to exhibit altered expression of circadian genes [20], more direct evidence of potential causality comes from loss-of-function studies. It has been shown that clock gene mutation or jet-lag paradigms disrupting circadian rhythmicity cause neurodegeneration and cognitive deficits [3]. For example, *Bmal1* disruption was recently found to increase amyloid burden in an AD mouse model [19].

Another emerging AD risk factor is dysregulated energy metabolism. Epidemiological evidence shows that type 2 diabetes (T2D) increases the risk of AD by 2–3-fold [21]. Furthermore, diabetes and obesity were found to aggravate amyloid pathology and cognitive impairments in AD mice [22,23,24]. Insulin can cross the blood–brain barrier into the brain, regulating energy homeostasis, cognition and behavior, and pilot studies suggest a functional relationship between AD and brain insulin resistance [25,26,27]. For example, analysis of post-mortem AD brains uncovered broadly impaired gene expression and kinase activation reminiscent of insulin resistance in T2D, prompting the investigators to refer to AD as type 3 diabetes [28]. Examination of insulin sensitivity ex vivo using post-mortem brain tissues further revealed a positive correlation of cognitive deficits in AD and insulin resistance [29]. Mitochondrial dysfunction is closely related to insulin resistance [30], and improving mitochondrial function has been shown to enhance insulin sensitivity [31]. There is also a strong link between mitochondria and AD. On the one hand, amyloids promote ROS production in mitochondria [21,32]; on the other, mitochondrial dysfunction in fact precedes disease manifestation and promotes pathogenesis [32]. Several dietary interventions, particularly intermittent fasting, have shown efficacies to bolster brain mitochondrial function and/or neuronal stress resistance, which correlates with robust anti-aging and anti-AD effects [33].

An important discovery from circadian research over the past two decades is the critical regulatory role of circadian clocks in energy homeostasis and physiological fitness [34]. Key metabolic genes in different tissues are controlled by the clock, and manipulating the clock, either by disrupting or enhancing clock functions, can have major consequences on energy metabolism [34,35]. Dietary interventions such as caloric restriction and time-restricted feeding have been shown to consolidate feeding and activity to a restricted time window, thereby increasing circadian amplitude in gene expression and metabolism [36,37,38]. Likewise, various small molecules have been reported to manipulate circadian rhythms and downstream physiology [39,40,41]. For example, we previously identified a natural compound called Nobiletin (NOB) capable of activating RORs and the circadian oscillator and promoting various metabolic and physiological functions in both young and aged mice [42,43,44,45]. NOB is a polymethoxylated flavonoid that is enriched in citrus peels and shows excellent safety and bioavailability profiles [46]. Functional studies have reported a wide variety of beneficial roles of NOB, including chemopreventive, anti-inflammatory and anti-oxidative efficacies in many disease and aging models [47,48,49,50]. In particular, growing evidence indicates neuroprotective effects of NOB in various rodent models for dementia, including AD [51,52]. In the current study, we aimed to investigate how NOB alters circadian rhythms, including sleep, and related pathophysiology in AD model mice.

## 2. Materials and Methods

### 2.1. Animals

Animal husbandry and experiments, using female mice, were approved by UTHealth Center for Laboratory Animal Medicine and Care (CLAMC; protocol # AWC-20-0058 and # AWC-18-0064) and were conducted in compliance with IACUC guidelines. The APP/PS1 double transgenic mice (JAX 034829) express a chimeric mouse/human amyloid precursor protein (Mo/HuAPP695swe) and a mutant human presenilin 1 (PS1-dE9). APP/PS1 mice were bred with B6C3F1/J (JAX 100010) in-house to create APP/PS1 and WT littermates. Mice were maintained under 12 h:12 h light:dark (LD) cycles unless otherwise noted. Zeitgeber time (ZT) 0 and 12 represent light on (7 am) and off (7 pm), respectively. Female mice were used for the current study. At 3–4 months of age, mice were treated with regular diets containing equivalent macronutrients with Purina 5053 with or without 0.1% Nobiletin (Research Diets, New Brunswick, NJ, USA). Animals continued with respective diet treatment and were sacrificed at 19–22 months of age.

### 2.2. Circadian Activity and Period Measurement

Female APP/PS1 mice at approximately 16 months of age were transferred into individual cages with running wheels (300 lux, room temperature at 23–25 °C, and humidity 38–45%). Mice were entrained for ~3 weeks in normal LD cycles, followed by 2–3 weeks in constant darkness (DD) to measure baseline free-running periods during DD. The circadian free-running period was estimated by using the CLOCKLAB software (Actimetrics, Evanston, IL, USA).

### 2.3. Noninvasive Piezoelectric Transducer Sleep/Wake Recording

Sleep and wake states were determined by using a noninvasive piezoelectric transducer sleep/wake recording system (Signal Solutions LLC, Lexington, KY, USA) as previously descried [42,53]. Briefly, animals (10–11 months old) were housed individually for 48 h prior to data recording in the Piezo system in normal LD cycles. The recording was conducted for five days while mice were maintained in this photoperiod condition with free access to food and water. Data were calculated and extracted by using Sleepstats software (Signal Solutions LLC, Lexington, KY, USA).

### 2.4. Metabolic Chamber Analysis

Systemic energy metabolism in APP/PS1 mice was analyzed by indirect calorimetry in CLAMS metabolic chambers as previously described [43]. All animals were housed in individual cages in normal LD cycles, and measurements of oxygen consumption and CO_2_ production were recorded every 8–12 min over the entire course of the experiment.

### 2.5. Glucose Tolerance and Cold Tolerance Tests

Glucose tolerance and cold tolerance were tested as previously described [42]. For glucose tolerance, after fasting overnight, the mice (10–11 months old) were injected intraperitoneally with glucose (1 mg/kg body weight). Tail vein blood glucose was measured at 0, 15, 30, 60, and 120 min using a glucometer. The area under the curve (AUC) was calculated by the trapezoid method. To assess cold tolerance, initial body temperature of WT and APP/PS1 (~12 months old) mice was measured by the insertion of a rectal probe thermometer (ThermoWorks, Salt Lake City, UT, USA). Individually housed mice were then placed in a 4 °C cold room and provided ab libitum access to their assigned diet and water. The core body temperature was monitored hourly for 6 h.

### 2.6. Real-Time PCR Analysis

RT-qPCR analysis was conducted as previously described with minor modifications [54,55]. Total RNA was extracted from frozen cortex tissue using TRizol (GenDEPOT, Katy, TX, USA). A total of 1 µg of extracted RNAs was used for cDNA synthesis. Gene expression was analyzed by using QuantStudio 7 (Applied Biosystems, Waltham, MA, USA). Data were analyzed using Prism 8 software (GraphPad). Primer sequences are listed in Table 1.

### 2.7. Western Blotting

Western blotting was performed as previously described with minor modifications [42]. Three frozen lysate samples per group were randomly chosen and 15 µg of proteins were separated on SDS-polyacrylamide gels. Proteins were transferred onto nitrocellulose membrane and detected by the following specific antibodies from BioLegend (San Diego, CA, USA): 6E10 (previously Covance SIG-39320) and anti-CTF (previously Covance SIG-39150).

### 2.8. Immunohistochemistry

Immunohistochemical analyses were performed as previously reported [56,57]. Briefly, mouse brain tissues were placed in 10% neutral buffered formalin overnight. Tissues were embedded with paraffin, sectioning and placed on slides. Deparaffinized sections were stained with 4G8 antibody (#800701, BioLegend, San Diego, CA, USA) for amyloid-β (Aβ). Staining was visualized by using HRP-specific DAB substrate kit with nickel (Vector Laboratories, Burlingame, CA, USA) and mounted in DPX mounting medium (Electron Microscopy Sciences, Hatfield, PA, USA). Image files were imported to ImageJ (NIH) for quantification. Approximately 4–6 slices per animal were used and Aβ was quantified using burden threshold.

### 2.9. Quantifications and Statistical Analysis

Results are presented as mean ± SEM unless otherwise stated. Data were analyzed using Student’s *t*-test, one-way ANOVA followed by post-hoc analysis using Tukey’s multiple comparison test or two-way ANOVA followed by post-hoc analysis using Tukey’s test as offered by GraphPad Prism. A value of *p* < 0.05 was considered statistically significant.

## 3. Results

### 3.1. Sleep Behavior and Circadian Free-Running Rhythmin Female APP/PS1 Mice and NOB Effects

**Figure 1:**
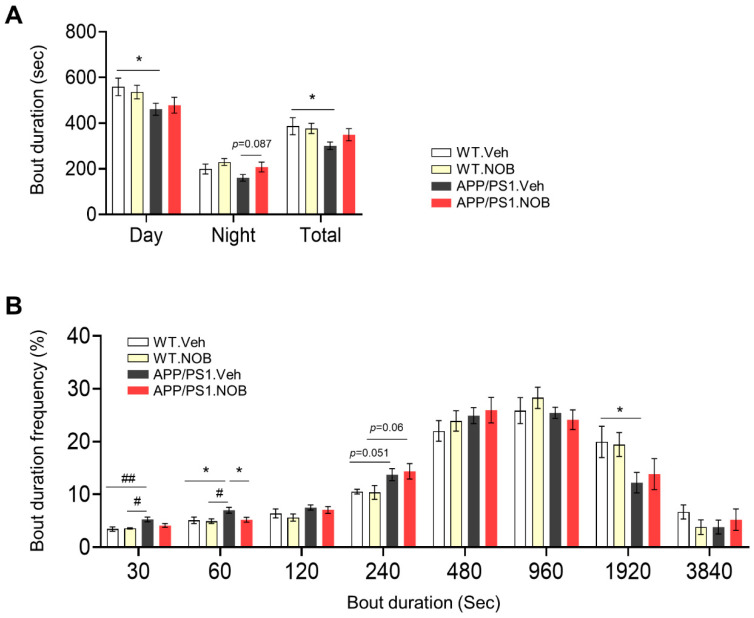
Sleep alterations in APP/PS1 mice and effects of Nobiletin (NOB). (**A**) Average of sleep bout duration (n = 6–10/each group). Data represented by mean ± SEM. * *p* < 0.05 unpaired Student’s *t*-test. (**B**) Histogram of sleep bout duration (n = 6–10/each group). Data represented by mean ± SEM. * *p* < 0.05 unpaired Student’s *t*-test. # *p* < 0.05, ## *p* < 0.01 one-way ANOVA with Tukey’s multiple comparisons test.

APP/PS1 is a widely used double transgenic mouse model for AD characterized by pronounced Aβ deposits [58]. In the current study, we used female WT and APP/PS1 mice considering that the female sex is one of the main non-modifiable risk factors for AD. We first conducted non-invasive piezo sleep assays to examine sleep cycles in these mice. Although the sleep amount was largely similar between genotypes under the control treatment (Figure S1A), APP/PS1.Veh mice showed reduced bout durations (Figure 1A) and correspondingly increased bout numbers (Figure S1B) relative to WT.Veh, suggesting impaired sleep consolidation and quality. NOB exhibited a trend to improve these parameters (Figure 1A and Figure S1A). A more detailed examination of bout duration (Figure 1B) revealed that the number of short sleep bouts with a duration of ~60 s was significantly reduced by NOB in APP/PS1 mice. Next, we carried out circadian wheel-running behavioral assays to evaluate circadian free-running rhythms under constant darkness. We did not observe statistically significant changes in behavioral period length (Figure S1C,D) or total activity (Figure S1E), although there may be a slight trend of period shortening in APP/PS1.Veh relative to WT.Veh, which was normalized by NOB (Figure S1D). Together, these results reveal changes in sleep behavior in APP/PS1 mice compared with WT and a trend of NOB to normalize it.

### 3.2. Effects of NOB on Systemic Metabolism in APP/PS1 Mice

**Figure 2:**
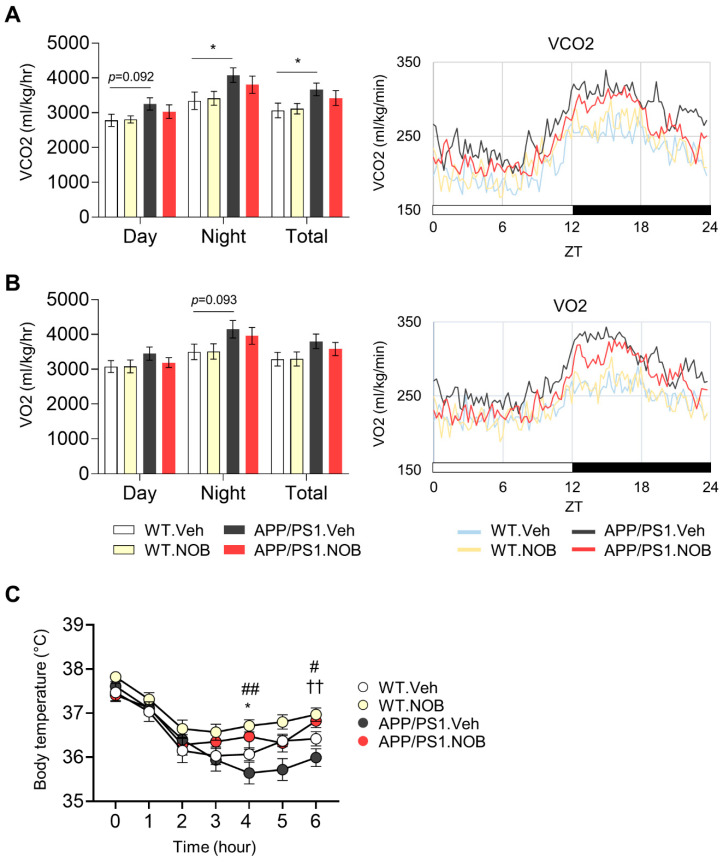
Nobiletin improves systemic metabolism in APP/PS1 mice. (**A, B**) Total average (left panels) and hourly average (right panels) of carbon dioxide production (VCO2) and oxygen consumption (VO2) using CLAMS metabolic chambers (n = 6–10/each group). Data represented by mean ± SEM. * *p* < 0.05 *t*-test. For the hourly average of VCO2: * *p* < 0.05 at the time points of 19.3 and 22.8 h, two-way ANOVA with Tukey’s multiple comparison test (APP/PS1.Veh vs. APP/PS1.NOB). For hourly average of VO2: * *p* < 0.05 at the time points of 13.5, 15, and 16.3 h, two-way ANOVA with Tukey’s multiple comparison test (WT.Veh vs. APP/PS1.Veh). (**C**) Cold tolerance test (n = 6–10/each group). Data represented by mean ± SEM. # *p* < 0.05, ## *p* < 0.01 unpaired student *t*-test (WT.Veh vs WT.NOB). * *p* < 0.05 unpaired student *t*-test (APP/PS1.Veh vs APP/PS1.NOB). †† *p* < 0.01 one-way ANOVA with Tukey’s multiple comparisons test (APPS/PS1.Veh vs. APP/PS1.NOB).

We next examined systemic metabolism in WT and APP/PS1 mice. Glucose tolerance tests did not show any significant difference between the groups (Figure S2A). In contrast, indirect calorimetry in metabolic chambers revealed significant effects on respiratory activities. Specifically, APP/PS1.Veh mice exhibited significantly elevated carbon dioxide production (VCO2) compared with WT.Veh (Figure 2A); in accordance, there was a trend of increase in oxygen consumption (VO2) and heat production (Figure S2B) compared to WT.Veh. Furthermore, monitoring of VO2 and VCO2 over the circadian cycle demonstrated strongly elevated levels of VO2 and VCO2 in APP/PS1.Veh at night-time (Figure 2A,B; see also figure legend), and NOB showed effects to partially normalize these levels in APP/PS1 mice, especially during the late dark phase. On the other hand, respiratory exchange ratio (RER) remained unchanged in the groups examined (Figure S2B,C).

Next, we conducted cold tolerance tests to investigate adaptive thermogenesis. APP/PS1.Veh mice showed significantly lower core body temperature compared to WT.Veh at 4 h and 6 h, and interestingly NOB strongly restored core body temperature in APP/PS1.NOB mice at 6 h (*p* < 0.01) compared to APP/PS1.Veh (Figure 2C). These results demonstrate systemic effects of NOB on mouse physiology, specifically respiratory activity and adaptive thermogenesis.

### 3.3. NOB Regulates Circadian Gene Expression in the Cortex

**Figure 3:**
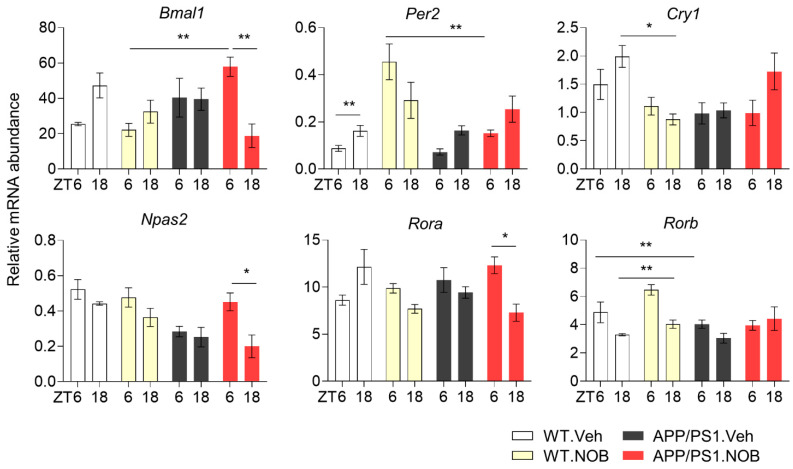
Effects of NOB on expression of core clock genes in WT and APP/PS1 mice. mRNA expression of core clock genes in cortex tissues was measured via real-time qPCR (n ≥ 3/each group). Mice were sacrificed at ZT6 and ZT18 under the LD condition. Data represented by mean ± SEM in bar graph. * *p* < 0.05, ** *p* < 0.01 two-way ANOVA with Tukey’s multiple comparisons.

Our previous study reported that NOB activated RORs in the core circadian oscillator to regulate clock and clock-controlled gene expression [43]. To examine NOB effects on the circadian oscillator at the molecular level, we measured clock gene expression focusing on the cerebral cortex, a tissue prone to Aβ deposition in AD. APP/PS1 mice displayed significantly altered clock gene expression patterns compared with WT mice (Figure 3), indicating circadian alteration in the cortex clock. Interestingly, expression of ROR target genes such as *Bmal1* and *Npas2* exhibited markedly increased diurnal changes in APP/PS1.NOB mice compared to APP/PS1.Veh. APP/PS1.NOB mice showed higher *Bmal1* expression at ZT6 compared with ZT18, suggesting a distinct temporal pattern from other groups. This alteration may be caused by NOB activation of RORs [43] (which directly regulate *Bmal1* transcription) and/or phase shifts in these mice. *Rora* also displayed a stronger diurnal alternation in APP/PS1 mice than *Rorb*, another *Ror* subtype that is expressed in the brain [59]. In comparison, expression of several other clock genes including *Clock*, *Per1*, *Cry2*, *Nr1d1*, and *Dbp* remained largely unchanged between groups (Figure S3). These results suggest altered circadian oscillators in the cortex of APP/PS1 mice, and NOB strongly modulates circadian time-dependent expression of several ROR target genes in the oscillator.

### 3.4. NOB Remodels Clock-Controlled Gene Expression in the Cortex

**Figure 4:**
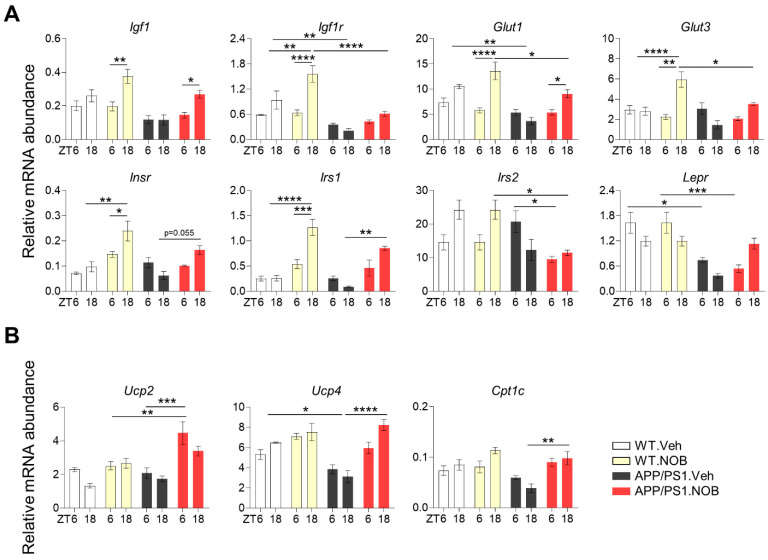
Nobiletin alters metabolic and mitochondrial gene expression in APP/PS1 mice. mRNA expression of (**A**) insulin signaling and glucose transporter-related genes and (**B**) mitochondrial thermogenic genes in cortex tissues was measured using real-time qPCR (n ≥ 3/each group). Mice were sacrificed at ZT6 and ZT18 under the LD condition. Data represented by mean ± SEM in bar graph. * *p* < 0.05, ** *p* < 0.01, *** *p* < 0.001, **** *p* < 0.0001, two-way ANOVA with Tukey’s multiple comparisons.

Circadian clocks are known to regulate metabolic and mitochondrial gene expression [34]; furthermore, energy metabolism, particularly the insulin pathway, and mitochondrial function have been implicated as modifying factors for AD [26]. We next examined key genes involved in these processes, many of which have been shown to be subjected to circadian control [18]. A number of genes involved in insulin signaling and metabolic homeostasis were found to be altered in APP/PS1 mice and normalized to varying degrees by NOB in the cortex (Figure 4A). For example, APP/PS1.Veh mice displayed lower levels of *Igf1r* and *Glut1* expression compared to WT.Veh. On the other hand, NOB rescued the expression of *Igf1*, *Glut1*, *Insr*, and *Irs1*. Of note, NOB enhanced the diurnal alteration of *Igf1*, *Glut1*, and *Insr* expression in both WT and APP/PS1 models.

We next examined neuronal mitochondrial genes involved in respiratory uncoupling (*Ucp2* and *Ucp4*) and fatty acid oxidation (*Cpt1c*) (Figure 4B). While there was a trend of decreased cortex expression of *Ucp4* and *Cpt1c* in APP/PS1.Veh compared to WT.Veh, NOB was able to significantly elevate expression of *Ucp2*, *Ucp4*, and *Cpt1c* in APP/PS1.NOB. Together, these results indicate a strong effect of NOB on metabolic gene expression in the cortex.

### 3.5. NOB Attenuates Amyloid Beta (Aβ) Plaque Deposition in APP/PS1 Mice

**Figure 5:**
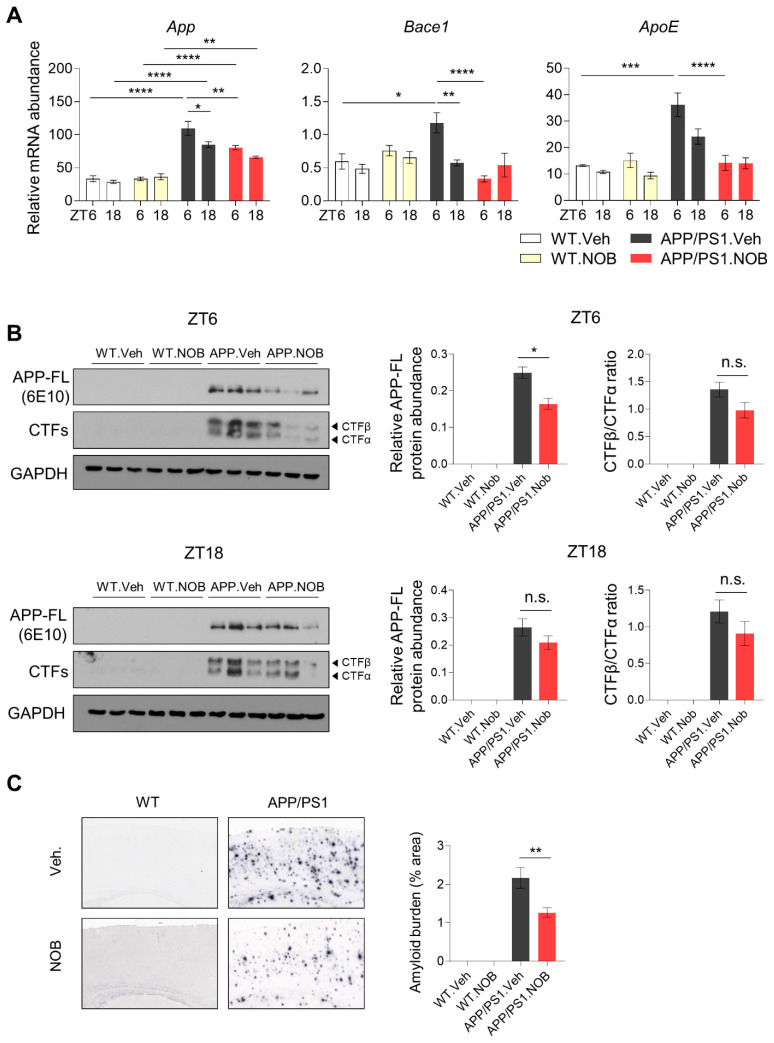
NOB modulates AD gene expression and ameliorates Aβ deposits in APP/PS1 mice. (**A**) mRNA expression of AD-related genes in WT and APP/PS1 mice. mRNA expressions of core clock genes in cortex tissues were measured using real-time qPCR (n ≥ 3/each group). Mice were sacrificed at ZT6 and ZT18 under the LD condition. Data represented by mean ± SEM in bar graph. * *p* < 0.05, ** *p* < 0.01, *** *p* < 0.001, and **** *p* < 0.0001. (**B**) Protein levels of APP-FL and CTFs were detected by using human 6E10 and carboxyl-terminal fragments (CTFs)-specific antibodies in cortex lysates at ZT6 and ZT18. GAPDH served as the loading control. Left panels: representative blot images. See Figure S4B for the whole blot images and size markers. Right panels: quantification of APP-FL and CTFs (n = 3/group). Data represented by mean ± SEM. * *p* < 0.05, unpaired Student’s *t*-test. (**C**) Immunohistochemistry of Aβ in APP/PS1 mice using the 4G8 antibody in the cortex (n = 6–11). Right panels: Quantification of amyloid burden (4–6 slices/mouse). Data represented by mean ± SEM. One-way ANOVA with Tukey’s multiple comparison test shows significant statistical difference between APP/PS1.Veh and APP/PS1.NOB (**, *p* < 0.01).

We first examined NOB effects on cortex expression of AD-related genes. As shown in Figure 5A, NOB reduced *App* mRNA levels in APP/PS1.NOB mice compared to APP/PS1.Veh, especially at ZT6 (*p* < 0.01), mitigating the transgene overexpression. In addition, *Bace1* and *ApoE* showed elevated expression in APP/PS1.Veh relative to WT.Veh, and NOB was able to strongly repress their levels in APP/PS1.NOB compared to APP/PS1.Veh at ZT6 (*p* < 0.0001 for all). Other examined AD-related genes, such as *Bace2*, *Scna*, *Scnb*, and *Lrp1*, did not show significant changes between APP/PS1.Veh and APP/PS1.NOB (Figure S4A). We next investigated levels of APP proteins and C-terminal cleavage products by Western blotting. As shown in Figure 5B and Figure S4B, the amounts of full-length APP proteins (APP-FL) were significantly reduced by NOB at ZT6, but not at ZT18, concordant with mRNA expression patterns in Figure 5A. The ratio of C-terminal cleavage products, CTFβ and CTFα corresponding to amyloidogenic and non-amyloidogenic pathways, respectively [60,61], was not significantly altered by NOB, although there may be a slight trend (Figure 5B). These results underscore the role of NOB in reducing APP protein expression in the cortex.

Finally, we investigated the effect of NOB on Aβ pathology in APP/PS1 mice. We stained Aβ plaque deposits using immunohistochemistry with the 4G8 antibody and observed significant Aβ plaque deposition in the cortex of APP/PS1.Veh mice, as expected (Figure 5C). Interestingly, the percent area of Aβ plaques in the cortex was strongly reduced by NOB treatment in APP/PS1 mice (*p* < 0.01), consistent with the above expression analyses. Taken together, these results illustrate a potent role of NOB in modulating AD gene expression and Aβ pathology in the cortex of female APP/PS1 mice.

## 4. Discussion

The current study aimed to characterize circadian and metabolic alterations in female APP/PS1 mice and investigate effects of the clock-modulating compound NOB. We show that sleep bout duration was reduced in female APP/PS1 mice, suggesting sleep fragmentation and reduced amplitude. NOB showed a trend to restore bout duration, but the effect did not reach statistical significance. NOB did not significantly alter the circadian free-running period or total activity in APP/PS1. Systemic metabolic characterization revealed that while glucose tolerance was largely unchanged irrespective of genotype or treatment, O_2_ consumption and CO_2_ production were increased in APP/PS1 mice relative to WT, resulting in a trend of elevated heat production under normal conditions. When subjected to cold challenge, APP/PS1 displayed impaired adaptive thermogenesis, and interestingly NOB was able to rescue it. This result falls in line with the previous observation that a mouse model of AD is susceptible to cold environment and exhibits impaired thermoregulation [62]. In our previous studies using naturally aged mice, we showed that NOB was able to increase sleep bout duration and improve glucose and other metabolic parameters to more significant degrees [42,63]. The relatively moderate systemic effects of NOB in APP/PS1 mice compared with aged mice may indicate more severe abnormalities in the former that are less amenable to manipulation, or alternatively may be attributable to technical factors such as differences in genetic background.

Importantly, when examining the cortex more specifically, we observed strong effects on core clock and clock-controlled output gene expression, and APP protein production and Aβ deposition were significantly alleviated by NOB in a circadian time-dependent manner, with more pronounced effects at ZT6. For example, diurnal expression of *Bmal1* and *Npas2*, both encoding positive circadian transcription factors in the core oscillator, was found to be significantly altered by NOB in APP/PS1 mice. These genes have been shown to be regulated by the ROR receptors and our previous analysis showed that NOB directly activates RORs [15,43]. Furthermore, several clock-controlled genes involved in insulin signaling and mitochondrial function are also modulated by NOB, including *Igf1*, *Igl1r*, *Glut1*, *Insr*, *Irs1*, *Ucp2*, *Ucp4*, and *Cpt1c*. These results are, on the one hand, consistent with prior studies showing their altered expression in AD models, particularly APP/PS1 [64,65,66]. For example, APP/PS1 mice have been found to express lower levels of GLUT1 in the hippocampus compared to WT, which negatively correlated with Aβ plaque burden [64]. Changes in the expression of these genes also suggest a regulatory role of mTOR signaling known to be involved in both AD and circadian rhythms [67,68,69,70]. On the other hand, we also observed marked effects of NOB to normalize expression patternsof these key genes; for example, NOB enhanced both the expression levels and diurnal alterations of *Igf1*, *Glut1*, and *Insr* in APP/PS1 mice. These effects suggest a metabolic regulatory function of NOB in the cortex, in accordance with previously demonstrated efficacy of NOB in peripheral tissues [42,43,48,71]. Finally, at both mRNA and protein levels, NOB was able to considerably reduce APP expression, likely contributing to the observed improvement in plaque pathology. *Bace1* and *ApoE* mRNA expression was also elevated in APP/PS1, consistent with previous findings [72], and NOB was able to normalize their levels. Together, these results on core clock, metabolic and AD genes in the cortex are consistent with a role of NOB to activate the circadian oscillator and modulate downstream gene expression, supporting a possible link between circadian physiology and AD.

Previous research has demonstrated many bioactive roles of NOB, including in various dementia and AD rodent models [47,49,51]. For example, NOB treatment of the 3xTg AD model mice harboring triple AD mutations was found to decrease the levels of soluble Aβ in the brain and improve cognitive performance [73]. Importantly, NOB is generally safe, and NOB-rich citrus extract has been applied in a small human study in conjunction with donepezil, an FDA-approved cholinesterase inhibitor, which led to improved cognitive performance in early-stage AD patients [74]. Supported by independent studies from many other labs, we discovered a novel function of NOB to enhance circadian rhythms which may serve as a unifying mechanism for the diverse beneficial effects [50]. A number of metabolic and physiological processes were found to be enhanced in both disease models and naturally aged mice, in a clock-dependent manner. As various pathogenetic mechanisms of AD, including gene regulation, sleep, and metabolism, are known to be regulated by the clock [3,25], understanding the role of circadian rhythms in AD progression may reveal important insights. To this end, the current study characterized the effects of NOB on these AD-related physiological processes in the commonly used APP/PS1 mice. Our results illustrate beneficial effects of NOB on circadian physiology, as well as gene expression and plaque deposition in the cortex, of female APP/PS1 mice, supporting the notion that circadian targeting by NOB may orchestrate distinct cellular processes relevant for AD pathology. Previously, several other studies have reported other clock-modulating small molecules that act on the circadian oscillator to improve AD-related pathophysiology and behaviors [75,76,77,78]. For example, it was shown that inhibition of REV-ERBs by the antagonist compound SR8278 in a highly aggressive AD mouse model (5xFAD) was able to promote amyloid uptake by microglia and consequently ameliorate plaque formation [76]. Since RORs and REV-ERBs are known to compete with each other in the secondary loop of the oscillator [15], these findings are consistent with our study where mice are treated with the ROR agonist NOB and highlight the secondary loop of the oscillator as a potential target for therapeutic development against AD.

There are several questions that await further studies. The detailed mechanisms, at both molecular and physiological levels, remain to be further investigated. For example, it is unclear how circadian oscillators and/or RORs control key genes to elicit the observed physiological and pathological effects. While we observed changes in metabolic processes, including thermogenesis and metabolic gene expression in the cortex, the specific roles of central and systemic metabolism in AD remain to be elucidated. Circadian clocks and NOB are also known to be involved in cellular mechanisms widely believed to contribute to AD pathogenesis, such as ROS/neuroinflammation [3,47]. For example, our previous studies showed that NOB optimizes mitochondrial respiration in skeletal muscle in aged mice, in part via effects on mitochondrial supercomplex formation [42]. It will be interesting to investigate whether NOB can modulate brain ROS production and neuroinflammation in AD mice via circadian pathways. Furthermore, we employed female mice for the current study. Traditionally, female mice are understudied in circadian research, in part due to a potential complication from estrous cycles. Only one third of recent studies of AD mouse models examined female mice [79]. However, AD is known to display a sexual dimorphism, with females accounting for appropriately two thirds of the patient population [80]. Currently, we are conducting studies using male mice in order to better understand the role of sex as a biological variable in AD. Finally, NOB has been shown to activate a broad spectrum of cellular pathways and many of these prior studies were conducted without consideration of circadian time [47,48]. It remains to be investigated whether the cellular effects emanate from circadian and ROR activation by NOB, or a non-circadian target/mechanism is involved. Particularly, future studies are needed to determine whether some of the effects reported herein may be attributable, at least in part, to a non-circadian mode of action by NOB.

In conclusion, our results provide new insights into the function of NOB to modulate circadian rhythms and physiology, which may contribute to the observed beneficial effect on amyloid pathology in female APP/PS1 mice. These results suggest a novel interventional strategy to target the circadian oscillator against AD progression.

## Figures and Tables

**Table 1:** Primer sequences for RT-qPCR.

	Forward (5′–3′)	Reverse (5′–3′)
*Clock*	CCTTCAGCAGTCAGTCCATAAAC	AGACATCGCTGGCTGTGTTAA
*Bmal1*	CCACCTCAGAGCCATTGATACA	GAGCAGGTTTAGTTCCACTTTGTCT
*Per1*	TTCGTGGACTTGACACCTCTT	GGGAACGCTTTGCTTTAGAT
*Per2*	ATGCTCGCCATCCACAAGA	GCGGAATCGAATGGGAGAAT
*Cry1*	CTGGCGTGGAAGTCATCGT	CTGTCCGCCATTGAGTTCTATG
*Npas2*	CAACAGACGGCAGCATCATCT	TTCTGATCCATGACATCCGC
*Rora*	GCACCTGACCGAAGACGAAA	GAGCGATCCGCTGACATCA
*Rorb*	GACCCACACCTACGAGGAAA	GTGATCTGGATGGCACACTG
*Nr1d1*	CATGGTGCTACTGTGTAAGGTGTGT	CACAGGCGTGCACTCCATAG
*Dbp*	CTGGCCCGAGTCTTTTTGC	CCAGGTCCACGTATTCCACG
*App*	AGCACCGAGAGAGAATGTCC	GCAAGTTCTTGGCTTGACG
*Bace1*	ACATTGCTGCCATCACTGAA	GCCTGGCAATCTCAGCATAG
*Bace2*	TGAGGACCTTGTCACCATCCCAAA	TGGCCAAAGCAGCATAAGCAAGTC
*ApoE*	ATTGCGAAGATGAAGGCTCT	CCACTCGAGCTGATCTGTCA
*Scna*	TGACAGCAGTCGCTCAGA	CATGTCTTCCAGGATTCCTTC
*Scnb*	GGAGGAGCTGTGTTCTCTGG	TCCTCTGGCTTCAGGTCTGT
*Lrp1*	ATTGAGGGCAAGATGACACA	CCAGTCTGTCCAGTACATCCAC
*Igf1*	TGGTGACCGGCTACGTGAAG	CAAAGTACATCTTTCCGGACC
*Igf1r*	ATCGCGATTTCTGCGCCAACA	TTCTTCTCTTCATCGCCGCAGACT
*Glut1*	AGCCCTGCTACAGTGTAT	AGGTCTCGGGTCACATC
*Glut3*	TAAACCAGCTGGGCATCGTTGTTG	AATGATGGTTAAGCCAAGGAGCCC
*Insr*	GACAGCCACCACACTCACACTTC	GTGCAGCTCCTCATCACCATATCG
*Irs1*	CCAGCCTGGCTATTTAGCTG	TTCTCTAGGAGCTGGGTGGA
*Irs2*	TCTTTCACGACTGTGGCTTCCTT	CACTGGAGCTTTGCCCTCTGC
*Lepr*	GTGTGAGGAGGTACGTGGTGAAG	CCGAGGGAATTGACAGCCAGAAC
*Ucp2*	ATGGTTGGTTTCAAGGCCACA	CGGTATCCAGAGGGAAAGTGAT
*Ucp4*	TCGAGACAAACAAGGAAGGGG	GACCAAGGGGTCATTCTCAGC
*Cpt1c*	GCAGGAGATCTCACCGACAT	CCCTGGAATCCGTGTAGTGT
*Gapdh*	CAAGGTCATCCATGACAACTTTG	GGCCATCCACAGTCTTCTGG

## Data Availability

The data presented in this study are available upon request to the corresponding author.

## Supplementary Materials

The following are available online at https://www.mdpi.com/article/10.3390/biom11071004/s1, Figure S1: Sleep and circadian behavior in WT and APP/PS1 mice. Figure S2: Glucose tolerance and systemic metabolic phenotypes related to respiration and heat production. Figure S3: mRNA expression of core clock genes in WT and APP/PS1 mice. Figure S4: mRNA expression of AD-related genes and APP protein levels in WT and APP/PS1 mice.
