# Supplementary material for: Effects of the Clock Modulator Nobiletin on Circadian Rhythms and Pathophysiology in Female Mice of an Alzheimer’s Disease Model
Source: Biomolecules. 2021 Jul 9;11(7):1004. doi: 10.3390/biom11071004 (PMC8301787; doi:10.3390/biom11071004)
Supplement: Supplementary file 1 [file biomolecules-11-01004-s001.zip › biomolecules-1269824-supplementary.pdf]

Supplementary Materials

Effects of the clock modulator Nobiletin on circadian rhythms and pathophysiology in female mice of an Alzheimer's disease model

Kim et al.,

This SM file contains 4 supplementary figures (Figure S1-S4) and figure legends.

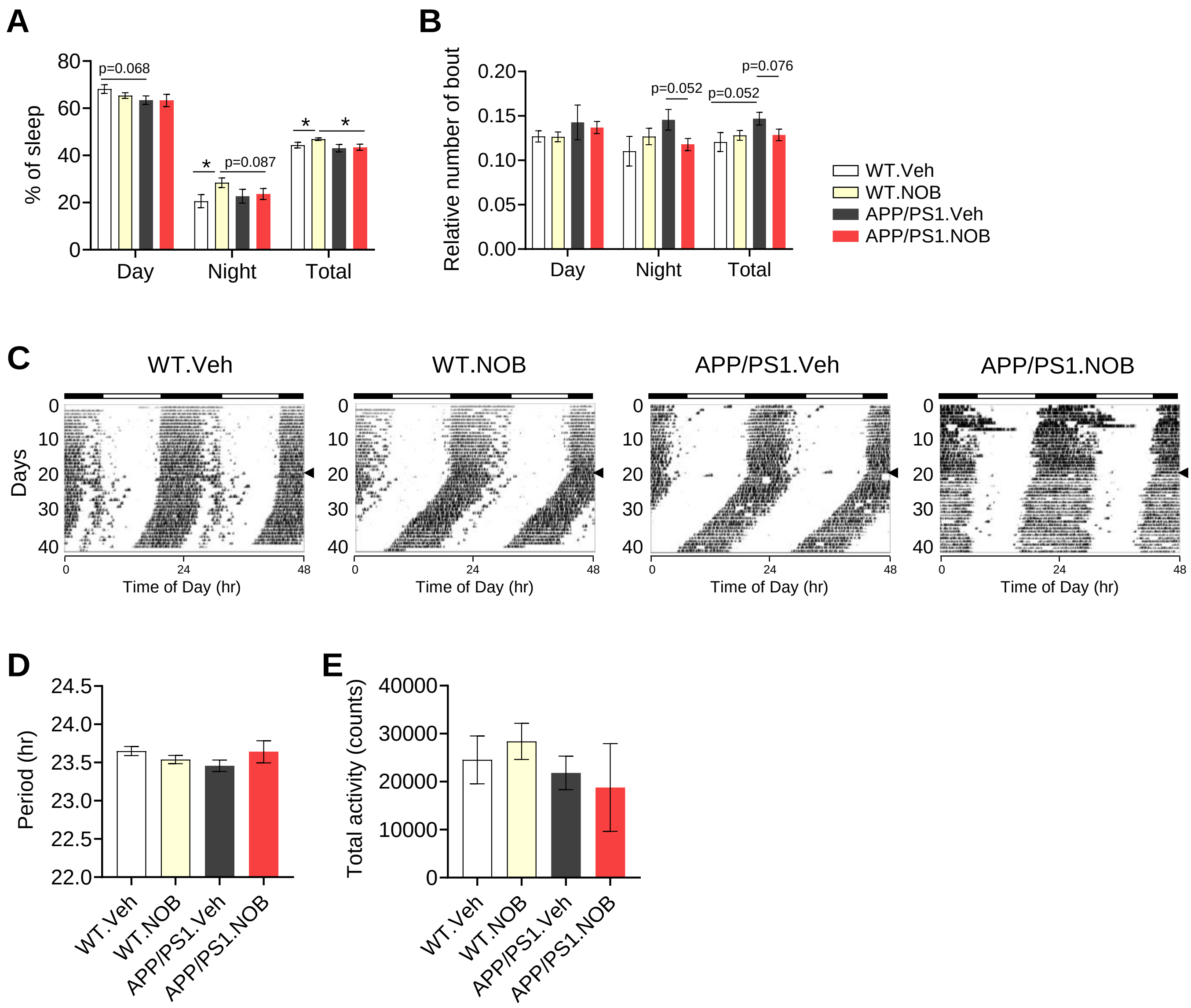

Figure S1. Sleep and circadian behavior in WT and APP/PS1 mice. (**A**) Percent of sleep (n=6-10/each group). Data represents mean ± SEM. * p<0.05 unpaired student t-test. (**B**) Relative number of sleep bout (n=6-10/each group). Data represents mean ± SEM. (**C**) Representative actograms of double-plotted wheel-running records obtained from WT and APP/PS1 mice on 12:12 light:dark cycles (LD) and in constant darkness (DD) (n=7-9/each group) as indicated by the white and black bars. Running activity is indicated by tick marks. Light transition from LD to DD is indicated by the arrow on the right. (**D**) Average circadian free-running periods and (**E**) total activity in constant darkness (DD) (n=5-9/each group). Data represents mean ± SEM.

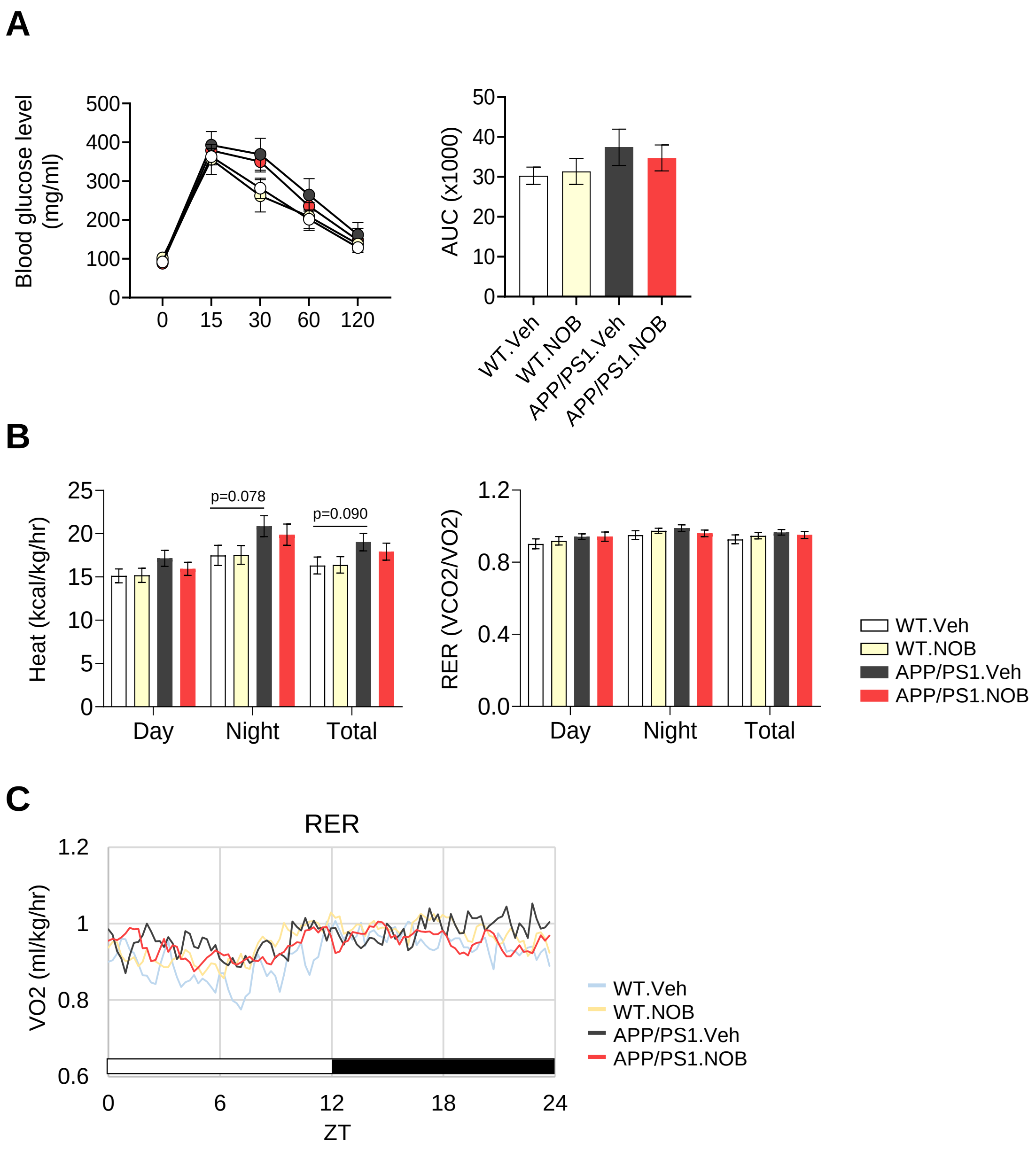

Figure S2. Glucose tolerance and systemic metabolic phenotypes related to respiration and heat production. (**A**) Glucose tolerance test and area under the curve (AUC) (n=6-10/each group). (**B**) Average of heat production and respiration exchange rate (RER) (n=6-10/each group). (**C**) Hourly average of RER (n=6-10/each group).

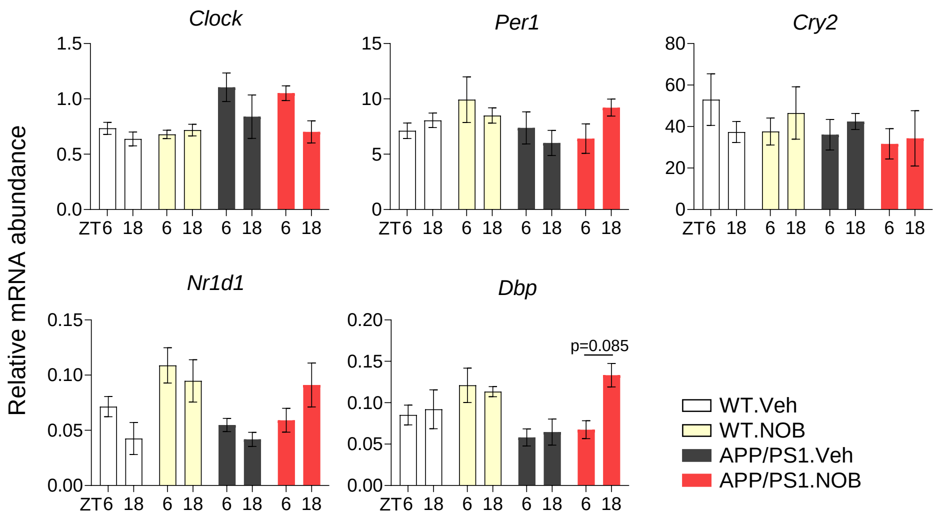

Figure S3. mRNA expression of core clock genes in cortex tissues from WT and APP/PS1 mice was measured by real-time qPCR (n ≥ 3/each group). Mice were sacrificed at ZT6 and ZT18 under the LD condition. Data presents mean ± SEM in bar graph. Two-way ANOVA with Tukey's multiple comparisons.

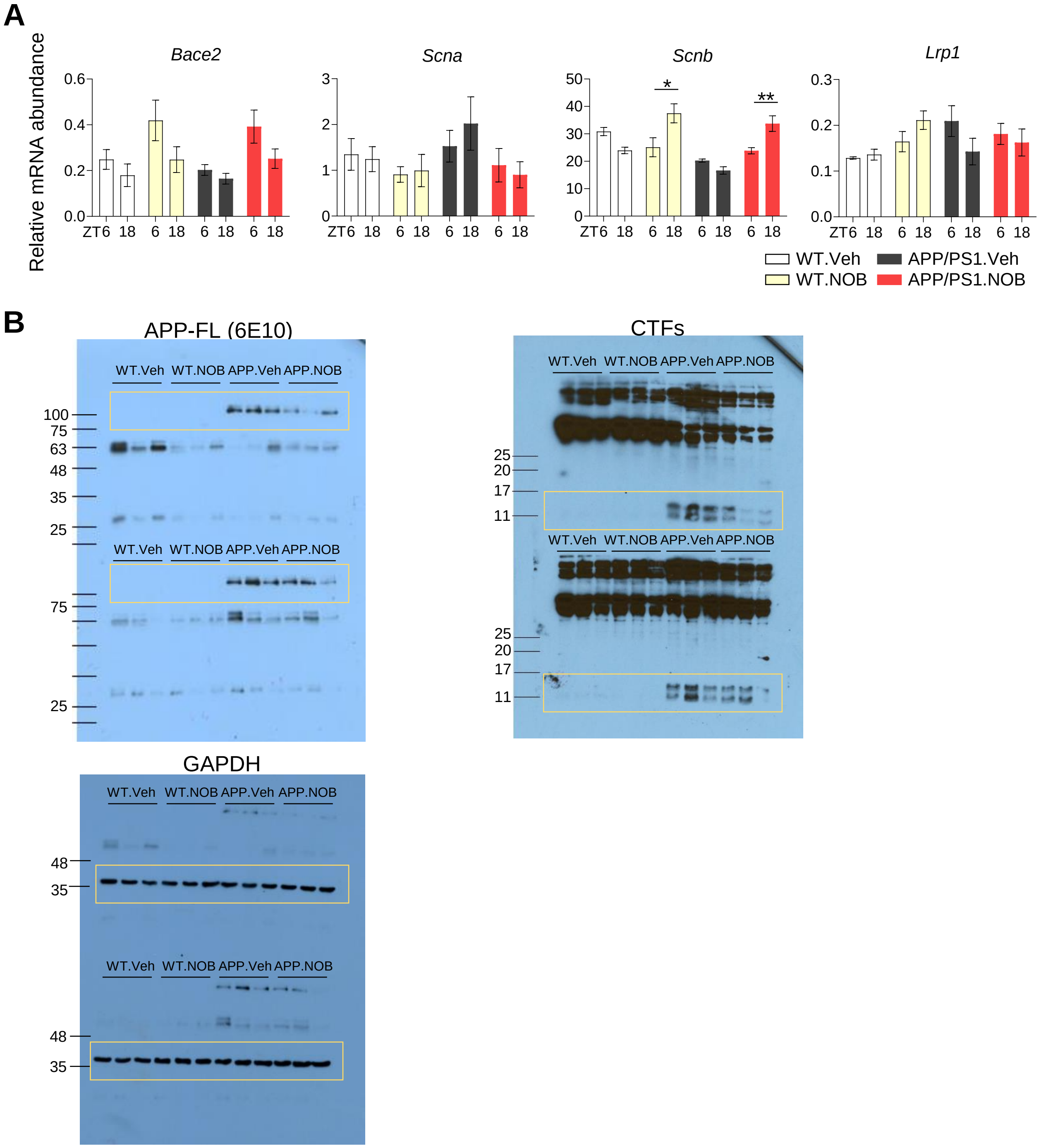

Figure S4. (**A**) mRNA expression of AD-related genes in in cortex tissues from WT and APP/PS1 mice was measured by real-time qPCR (n ≥ 3/each group). Mice were sacrificed at ZT6 and ZT18 under the LD condition. Data presents mean ± SEM in bar graph. (**B**) Original Western blot images of APP-FL (6E10), CTFs, and GAPDH. Top and bottom halves correspond to ZT6 and ZT18 respectively. Representative images in Figure 5B are indicated in yellow outline. Molecular size markers are denoted by numbers (in kDa).
